# Gut–Skin Axis: Current Knowledge of the Interrelationship between Microbial Dysbiosis and Skin Conditions

**DOI:** 10.3390/microorganisms9020353

**Published:** 2021-02-11

**Authors:** Britta De Pessemier, Lynda Grine, Melanie Debaere, Aglaya Maes, Bernhard Paetzold, Chris Callewaert

**Affiliations:** 1 Center for Microbial Ecology and Technology, Ghent University, Coupure Links 653, 9000 Ghent, Belgium; britta.depessemier@ugent.be (B.D.P.); melanie.debaere@ugent.be (M.D.); aglaya.maes@ugent.be (A.M.); 2 Department of Head & Skin, Ghent University, Corneel Heymanslaan 10, 9000 Ghent, Belgium; lynda.grine@ugent.be; 3 S-Biomedic, Turnhoutseweg 30, 2340 Beerse, Belgium; bernhard.paetzold@sbiomedic.com

**Keywords:** skin microbiome, gut dysbiosis, atopic dermatitis, acne vulgaris, psoriasis, dandruff, skin cancer, rosacea, wound healing, dietary, probiotics

## Abstract

The microbiome plays an important role in a wide variety of skin disorders. Not only is the skin microbiome altered, but also surprisingly many skin diseases are accompanied by an altered gut microbiome. The microbiome is a key regulator for the immune system, as it aims to maintain homeostasis by communicating with tissues and organs in a bidirectional manner. Hence, dysbiosis in the skin and/or gut microbiome is associated with an altered immune response, promoting the development of skin diseases, such as atopic dermatitis, psoriasis, acne vulgaris, dandruff, and even skin cancer. Here, we focus on the associations between the microbiome, diet, metabolites, and immune responses in skin pathologies. This review describes an exhaustive list of common skin conditions with associated dysbiosis in the skin microbiome as well as the current body of evidence on gut microbiome dysbiosis, dietary links, and their interplay with skin conditions. An enhanced understanding of the local skin and gut microbiome including the underlying mechanisms is necessary to shed light on the microbial involvement in human skin diseases and to develop new therapeutic approaches.

## 1. Introduction

The skin epidermis, along with its appendage structures, such as sweat and sebaceous glands, provide a total skin surface of about 25 m${}^{2}$ and is one of the largest epithelial surfaces for interaction with microbes [1]. The skin is a first-line barrier from the outer environment, continuously interacting with it. The gastrointestinal (GI) tract is one of the largest interfaces (30 m${}^{2}$) between the host and its environment [2]. About 60 tons of food is estimated to pass through the gut in a lifetime, all of which have a big impact on human health [3]. Both the gut and skin are immensely immersed with microbiota as it is estimated that the skin has about 10${}^{12}$ microbial cells while the gut accounts for 10${}^{14}$ microbial cells [4,5]. The microbiota point to the assemblage of specific microorganisms that are present within a defined environment. The emergence of next-generation sequencing in the past decade has provided unprecedented insights into microbiome composition, both on skin and in the gut. The microbiome refers to the genomes present in a certain environment, meaning the accumulation of all their genetic material (i.e., DNA and RNA). Both organs are characterized by a low microbial diversity at the phylum level but high diversity at the species level [6]. The microbiome provides a multitude of benefits to the host, such as shaping the immune system, protecting against pathogens, breaking down metabolites, and maintaining a healthy barrier [3].

The immuno-modulating potential of the microbiome on distant organ sites is an expanding research field. Especially the influence of the gut microbiome on distant organs, such as the lung, brain, and skin, have created the following areas of research: gut–lung axis, gut–brain axis, and gut–skin axis [7]. The innate and adaptive immune systems alter the microbial composition; however, the local microbiome can also modulate the immune system. The underlying mechanisms of how the gut microbiome alters the skin’s immune system, and vice versa, are currently being investigated. Several skin pathologies pose as gut comorbidities. Several studies have demonstrated the bidirectional link between gut dysbiosis and skin homeostasis imbalances, with a particular role of gut microbiota dysbiosis in the pathophysiology of multiple inflammatory diseases [8,9,10].

A summary of recent findings in the skin and gut microbiome in multiple skin disorders is given in this review, highlighting some potential mechanisms underlying the gut–skin axis.

## 2. Skin Versus Gut Barrier

The gut and skin barrier share surprisingly many features. The gut and skin are highly analogous to each other in purpose and functionality. Both organs are highly innervated and vascularized, as they are both essential for immune and neuroendocrine function [11]. The gut–skin axis results from this resemblance [11]. The inner surface of the gut and the outer surface of the skin are both covered by epithelial cells (ECs) which have direct contact with the exogenous environment [12]. This way, the immune system is continuously primed to distinguish between harmful and beneficial compounds. Immune cell priming starts early on in life and forms the basis of tolerance, a crucial concept hypothesized to be flawed in several autoimmune disorders [13]. ECs maintain an important link between the internal body and the external environment. They act as a first line of defense, preventing the entry of microorganisms [12]. Keratin, which is present in the stratified squamous epithelium of the skin, presents a formidable physical barrier to most microorganisms [14]. In addition, this compound makes the skin resistant to weak acids and bases, bacterial enzymes, and toxins [15]. Mucosae provide similar mechanical barriers, as it comprises a glycoprotein layer on top of the epithelium in which commensal bacteria reside [16,17]. The epithelial membranes produce protective chemicals that eliminate microorganisms [18]. The skin acidity (pH of 5.4 to 5.9) creates an inhospitable environment for potential pathogens and inhibits bacterial growth [19]. Sebum produced by the sebaceous glands acts as a seal for hair follicles and contains several antimicrobial molecules as well as specific nutritional lipids for beneficial microorganisms [20,21]. Meanwhile, in the digestive system, saliva and lacrimal fluid contain lysozyme, followed by the stomach mucosae that secrete strong acid and protein-digesting enzymes [22]. In addition, mucus traps microorganisms that enter the digestive and respiratory tract [23].

The second line of defense are the antimicrobial peptides (AMPs), phagocytes, and innate lymphoid cells (ILCs) [24]. These two first lines of defense form the innate immune system [23]. AMPs produced by keratinocytes, such as cathelicidin and psorasin, provide an effective barrier function to the skin [25,26]. The serine protease Kallikrein 5 (KLK5) cleaves cathelicidin into active peptides, such as LL-37 [27]. Compared to the skin, the composition of the intestinal epithelial barrier varies throughout the gastrointestinal tract. The proximal part of the gastrointestinal tract, the mouth and esophagus, is analogous to the skin, covered by multiple layers of squamous epithelium, which is cleansed by mucus from salivary and other glands [28]. The remaining part of the digestive tract includes a single layer of active cells, e.g., goblet cells (mucus secretion), enteroendocrine cells (hormone secretion), enterocytes or colonocytes (absorption), etc. [29,30]. The intestinal epithelium constitutes a single layer of enterocytes or colonocytes, and its barrier integrity is protected by the immune system. The absorptive functionality of the enterocytes in the small intestine ensues a discontinuous layer of mucus with fewer goblet cells [31]. Paneth cells are enriched in the crypts of the small intestine that secrete AMPs, which integrate in the complex mucus layer [32].

Microbial-associated molecular patterns (MAMPs) are sampled through antigen uptake by membranous (M) cells and goblet cells to dendritic cells (DCs), together with direct transepithelial luminal DCs. Microbial signals are sensed by RORγt innate lymphoid Cclls (group 3 ILCs) that produce interleukin-17 (IL-17) and IL-22 [33]. The latter acts directly on the intestinal epithelial cells (IECs) and activates damage repair mechanisms, AMPs, and mucin genes [34]. Plasma cells in Peyer’s patches, which are stimulated by DCs, produce IgA in the lamina propria in a T cell-independent manner [35,36]. The large intestine on the other hand contains a thick, continuous mucus layer to compartmentalize the microbiota, with IgA and AMPs having a secondary role [17]. The control of immunological processes within mucosal tissues is dependent on the interaction between ECs and DCs, as both cell types are involved in the sensing and sampling of antigens [12]. In the skin and intestine, pathogens are sampled via M cell-independent mechanisms [37]. The only DCs that are found within the epidermis are the Langerhans cells (LCs) [12].

Other similarities between the gut and skin tissues is the high cellular turnover rate, which inhibits adherence and infection by the colonizing microbiome [38,39]. The skin and the gut are the two major niches that host prokaryotic and eukaryotic symbiotic microorganisms [40,41]. However, the resident microbiota are frequently involved and play a crucial role in the pathogenesis of several diseases [42]. Both tissues are very responsive to stress and anxiety, as they face similar challenges. Remarkably, diseases such as inflammatory bowel Disease (IBD) and psoriasis comprise an epithelial barrier dysfunction and an increased epithelial cellular turnover rate. The increased permeability of the epidermal skin and intestinal barrier is due to the augmented interaction of allergens and pathogens with inflammatory receptors of immune cells. Both diseases have an analogous immune response and involve phagocytic, dendritic, and natural killer cells along with a range of cytokines and AMPs that induce a T cell response [43]. In addition, both diseases are characterized by dysbiosis in the microbiome composition that covers the respective interface linings [43].

The gut microbiome is the largest endocrine organ, producing at least 30 hormone-like compounds, e.g., short chain fatty acids (SCFAs); secondary bile acids; cortisol; and neurotransmitters such as gamma-aminobutyric acid (GABA), serotonin, dopamin, and tryptophan. Certain members of the gut microbiome respond to hormones secreted by the host [44]. The hormone-like pleiotropic compounds that are produced by the gut microbiome are released into the bloodstream and can act at distant organs and systems, such as the skin [44]. Numerous studies provided evidence for a profound bidirectional link between gastrointestinal health and skin homeostasis through modification of the immune system [45,46,47]. Modulation of the immune system occurs primarily through the gut microbiota. However, commensal skin microbiota are evenly essential for maintenance of the skin immune homeostasis [48]. Both the intestine and the skin host diverse bacterial, fungal, and viral species that maintain symbiosis with the human habitat. Disrupting this balance might lead to an impaired barrier function. Skin homeostasis recovery after disturbance or stress through gut microbiota enacts on both innate and adaptive immunity.

## 3. Skin and Gut Microbiome Involvements

The skin is the largest and most external barrier of the body with the outer environment. It is richly perfused with immune cells and heavily colonized by microbial cells, which in turn train the immune cells and determine the well-being of the host [49]. The skin microbiome has gained significant attention in recent years in dermatology, skin disorders, and its connection and influence on the immune system. Many skin conditions are associated with an imbalance in the skin microbiome (Table 1). More and more studies have shown enriched pathogens and microbiota that are associated with skin conditions, some of which are obvious and others more surprising. It is nonetheless difficult to determine whether the altered skin microbiome is a cause or consequence of the skin disorder.

The intestinal tract harbors a diverse collection of bacteria, fungi, and protozoa [50]. Many of these microorganisms are essential for metabolic and immune function, as they metabolize indigestible complex polysaccharides into essential nutrients such as vitamin K and B12, butyrate, and propionate [51,52]. The latter have a positive effect on the epithelial barrier integrity. Intestinal barrier integrity plays a crucial role in protecting microbiota from entering the systemic circulation and in avoiding inflammation in the gut. Diet can have a vital role in the maintenance of particular skin pathologies, when those food ingredients impair the intestinal barrier, which leads to gut bacteria entering the bloodstream.

Lifestyle factors such as diet and hygiene have a determining impact on the tolerance of the immune system to commensal microbiota, which in combination with genetic susceptibility, leads to microbial dysbiosis and disease. For instance, a Western diet has been associated with the development of numerous immune-mediated inflammatory diseases (IMIDs), such as rheumatoid arthritis, psoriasis, and atopic dermatitis (AD). Similarly, the hygiene hypothesis has been linked to the development of Th2-mediated diseases such as asthma and atopic dermatitis. The hygiene hypothesis implies that a reduced exposure to microbes through modern health practices can lead to increased inflammatory diseases in the urbanized society [53]. An overly hygienic lifestyle prevents microbial stimulation and can cause an atopic Th2-skewed response. People living in non-urbanized environments (indigenous people and farming environments) are usually not characterized by inflammatory diseases [54,55]. The mechanism of the Western diet, or high-fat diet (HFD) relies on the resulting intestinal dysbiosis, leading to an increase in the ratio of Firmicutes to Bacteroidetes. The mechanism for this phenomenon is outlined by Guo et al., who found that, in mice, HFD leads to a decreased release of AMPs in the small intestine, which is followed by changes in the composition of the gut microbiota and subsequent alterations in serological inflammatory cytokine levels [56].

Other nutritional components such as glycoalkaloids, alpha tomatine, and capsaicin, which are characteristic for the nightshade family legumes, have been associated with intestinal permeability [57]. In a similar manner, gluten can have an impact on skin health. Coeliac disease and gluten sensitivity have been linked with several skin conditions [58]. Dermatitis herpetiformis is a cutaneous manifestation of celiac disease, and patients can clear up skin rashes when shifting to a gluten-free diet for several months up to several years. The rashes generally return when patients resume gluten consumption [59]. Other allergic and autoimmune diseases, including psoriasis, have been associated with gluten intolerance [58]. Similarly, the strong association between atopic dermatitis and food allergy demonstrates the importance of food underlying the gut–skin axis [60].

However, the gut–skin axis not only is governed by diet but also acts bidirectionally. Skin exposure to ultraviolet B (UVB) and therefore indirectly to serum vitamin D levels increase the α and β diversity of the gut microbiome [61]. Bacteria from several families were enriched, and the serum vitamin D levels were correlated with the relative abundance of *Lachnospira* and *Fusicatenibacter* genera [61]. Moreover, food allergies may result from an impaired skin barrier: atopic dermatitis sensitizes to peanut allergy due to epicutaneous peanut protein exposure in household dust, leading ultimately to immunoglobulin E (IgE)-mediated mast cell expansion in the gut [62,63]. More specifically, the duodenum and oesophagus act as reservoirs for IgE+ B lineage cells [64].

**Figure 1:**
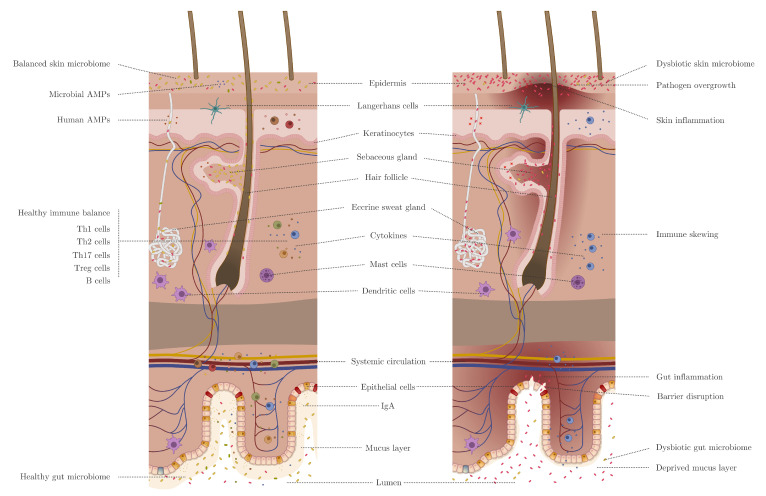
Inflammatory and microbial influences between the gut and skin for a healthy state (**left**) and a dysbiotic state (**right**): The intestinal and epidermal barriers are connected through the systemic circulation (blood and lymph) and are visualized here together in a simplistic manner. The dysbiotic state is characterized by an impaired gut barrier (imbalance in gut microbiome, reduced mucus layer, reduced IgA secretion, barrier disruption, intestinal permeation into the bloodstream, and gut inflammation) and an impaired skin barrier (imbalance in skin microbiome, reduced human and microbial antimicrobial peptides (AMP) production, skin rashes/thickening/lesions, and skin inflammation). Gut and skin dysbiosis are connected through an immune imbalance (Th2 skewing in this example), whereas crosstalk can be bidirectional.

Dysbiosis in the gastrointestinal system is quite often linked to inflammatory diseases (Table 2) [8,9,10]. Gastrointestinal disorders are associated with certain dermatoses, for instance, 7–11% of patients with IBD also suffer from psoriasis [65]. The connection between the skin and gut seems to be mediated by the host immune system. The interaction of the microorganisms and the host immune system is important to maintain the skin homeostasis. The gut–skin axis may be viewed as an integral part of the gut–brain–skin axis, elegantly described by Arck et al. and by Bowe and Logan [7,66]. Table 3 lists neurotransmitters that are produced by intestinal microbiota that might cross the intestinal barrier, enter the bloodstream, and instigate systemic effects (Figure 1) [67,68]. In addition, SCFAs, such as butyrate, acetate, and propionate, are fermentation products derived from undigested polysaccharides by intestinal bacteria (e.g., *Bacteroides*, *Bifidobacterium*, *Cutibacterium*, *Eubacterium*, *Lactobacillus*, and *Prevotella*) [69]. These SCFAs, especially butyrate, enhance epithelial barrier function and decrease the permeability of the intestinal barrier [70]. However, the SCFA quantity that enters the bloodstream is dependent on the individual fiber intake, the microbial fermentation rate, and the amount of colon absorption. All these compounds, which are derived from the gut, could all interact with skin receptors and could directly affect the skin or modify the skin’s commensal bacteria. Further research is needed to reveal if a clinical significant amount of SCFAs is reached in the bloodstream to affect the skin [11]. The studies from Table 3 support that the gut and skin interact with one another via the diet, microbial metabolites, the neuroendocrine pathways, and the central nervous system.

Here, we provide an overview of nine common skin disorders and their respective pathophysiology, and the body of knowledge regarding their skin microbiome alterations, imbalanced gut microbiome, and/or relation to a specific diet.

## 4. Acne Vulgaris

### 4.1. Acne Vulgaris Pathophysiology

Acne vulgaris is a multifactorial disease with the main drivers being the skin microbiome composition, the hormonal and immunological state of the host, sebum production, diet, FoxO1 deficiency, hormonal disorders, and deregulation of insulin-like growth factor. Acne vulgaris is the most common skin disorder in the Western world; it can affect 79% or 95% of the Western adolescent population [147]. It is surprisingly absent in hunter–gatherer communities and communities that live a traditional, non-Western lifestyle [147]. The immunology contribution is shaped by the innate immunity, adaptive immunity, and the T helper 17 (Th17) pathway [148,149]. However, the clinical relevance of the Th17 pathway in this disease still has to be evaluated as CD4+ IL-17-producing T cells were also found next to non-inflamed sebaceous glands [150,151]. A very different axis in the development of acne vulgaris is the insulin levels and insulin-like growth factor (IGF) concentrations. Some studies reported that IGF deficiency can protect from acne vulgaris [152]. A more complicated interplay with diet and other drivers in acne vulgaris is likely [153,154]. Currently, the main therapeutic approach includes antibacterial compounds, retinoids, or comedolytic actives. The diversity in mode of actions make it obvious that the underlying disease has many contributors [155].

### 4.2. Acne Vulgaris Skin Microbiome

Acne vulgaris is a widespread skin disease that usually affects sebaceous skin areas. The pathology and associated skin microbiome dysbiosis has been linked with certain strains of *Cutibacterium acnes*. The suggested factors range from sebum induction, direct immune system stimulation, diversity of the *C. acnes* population, porphyrin production, mobile genetics elements, and associated CRISPR/CAS loci to the production of SCFAs [156,157,158,159,160,161,162]. Many reviews on this topic are published, with the most recent being by Brüggemann [163]. Despite many years of research, the precise interdependence and choreography of pathogenic events in acne remain unclear. The main challenge is to distinguish between health- and disease-associated *C. acnes* strains. Multiple observational studies have been published on this subject and came to slightly varying conclusions. However, most of these studies overlap in the finding that Clade II strains are health-associated, while there is dispute on the functional reasoning behind this [164]. Some studies suggest that Clade IA strains are disease-associated [72]. However, this clade is also the most widespread in healthy skin. This leads to the search for pathogenicity-defining biomarkers in the subpopulation of Clade IA. Multiple biomarkers are suggested, such as mobile genetic elements, linoleic acid isomerase activity, porphyrin production, or cell adhesion. The search for an efficient biomarker that distinguishes acne-associated strains in Clade IA from health-associated strains is of particular interest as these strains have an evolutionary advantage over strains from Clade II, as demonstrated by their wide distribution. Additionally, Clade IA strains produce high levels of the antioxidant RoxP, which might be highly beneficial in protecting the host during the normal aging process [165]. One aspect that is often undervalued is the interplay of different *C. acnes* strains. A recent hypothesis stated that, instead of individual strains, the *C. acnes* strain diversity is a distinguishing driver of health and disease [166]. A limited number of studies have tested the potential use of probiotics (*Lactobacillus*, *Bifidobacterium*) to counteract the adverse effects of antibiotic treatments and as an alternative treatment for acne vulgaris [117]. These theories are promising and are currently further investigated [167].

### 4.3. Gut Microbiome and Diet Implications in Acne Vulgaris

Already many decades ago, a connection between the gastrointestinal part and acne vulgaris was suggested and followed by a first study in 1961 using oral *Lactobacillus* supplements [66]. Later, this was substantiated by a trial showing a strong association of diet and acne vulgaris [106]. Finally, in 2018, a study showed that acne vulgaris patients actually have a distinct gut microbiome composition [168]. Acne patients have decreased diversity of the gut microbiota with lower abundance of Firmicutes and increased levels of *Bacteroides*. Generally, *Clostridium*, Clostridiales, Lachnospiraceae, and Ruminococcaceae were depleted in the acne cohort. While a distinct difference between the acne cohort and the healthy controls was observed in this study, no correlation or distinctive biomarker was found correlating with acne severity. Multiple studies have been performed looking at the oral supplementation of probiotics in acne vulgaris [117,169,170]. The available results look promising, but a high heterogeneity in the provided products as well as shortcomings in the study design do not yet allow a final verdict on the efficacy of oral probiotic treatments in acne vulgaris. HFD contains a large quantity of saturated fats and a high glycemic load, which is strongly correlated with acne vulgaris [60,147]. The hypothetical cause is a disturbed nutrient signaling with an uncontrolled stimulation of sterol regulatory element-binding protein 1 (SREBP-1) and an increased synthesis of fatty acids and triglycerides in the sebum, which stimulates the growth of *C. acnes* [171]. While the gastrointestinal microbiome is only one of many factors contributing to acne, it has an undeniable impact on the skin condition in acne vulgaris. Until now, the exact mechanism is unclear but it is tempting to speculate a general influence of the gut microbiome on the immune system.

## 5. Atopic Dermatitis (AD)

### 5.1. AD Pathophysiology

Atopic dermatitis is the most common inflammatory skin disease (7% of adults and 15% of children). It is an inflammatory skin disorder characterized by barrier dysfunction, chronic inflammation, and microbial dysbiosis on skin [172]. AD is also influenced by host genetics and environment [173]. The inflammation is driven by a Th2 cytokine pathway, with the cytokines IL-4 and IL-13 playing an important role [174]. These play a central role in type 2 inflammation, not only for atopic dermatitis but also for several other allergic diseases. The IL-4 and IL-13 cytokines are involved in skin barrier disruption, decreased skin lipid metabolism, and inhibited antimicrobial peptide synthesis [175]. These conditions promote the growth and pathogenesis of *Staphylococcus aureus* [176]. The understanding of this disease has improved a lot in recent years. There is nonetheless still a large unmet need for long-term disease control.

Up to 30% of Caucasians have a mutation of the filaggrin gene, which codes for a crucial protein regulating the epidermal homeostasis [177]. Filaggrin is affected in both lesional and non-lesional skin.

Mild atopic dermatitis can be treated with skin moisturizers, topical corticosteroids, antihistamines, immunosuppressants, and phototherapy [178]. Twenty to thirty percent of patients have moderate-to-severe atopic dermatitis. For these patients, many biologics are in development or have been developed targeting the Th2 axis, such as IL-4, IL-13, IL-31, OX40, IL23p19, IL-5RA, and janus kinase (JAK)-inhibitors (which target several key AD cytokines) [179,180]. So far, only dupilumab has been shown to improve moderate-to-severe atopic dermatitis and subsequently approved [181].

### 5.2. Skin Microbiome in AD

Lesional skin in AD is commonly characterized by a low bacterial diversity [182]. The relative abundance of *Staphylococcus aureus* and *Staphylococcus epidermidis* is increased, while a decrease in *Cutibacterium*, *Corynebacterium*, *Streptococcus*, *Acinetobacter*, *Prevotella*, and *Malassezia* is found [80,83]. Specifically, *S. aureus* has long been associated with the skin pathology [183]. *S. aureus* has been found in higher relative and absolute abundances on lesional skin compared to nonlesional skin [184]. *S. aureus* was found more abundantly on AD skin compared to healthy controls [80,81]. The relative abundance of *S. aureus* is correlated with disease severity [82]. Colonization by *S. aureus* may play a critical role in perpetuating skin inflammation through the development of Th2 cells induced by peptidoglycan [185]. These are common cell components of *S. aureus* strains. The barrier disruption as well as reduced levels of ceramide cause the peptidoglycan to penetrate into the skin [185]. The peptidoglycan of *S. aureus* can induce human cathelicidin LL-37 and vascular endothelial growth factor (VEGF) expression in keratinocytes, with VEGF production being amplified by subsequent IL-13 overproduction [186]. *S. aureus* and its enterotoxins as such trigger inflammation through direct infection of the keratinocytes [187]. Due to the impaired barrier, AD lesions are also susceptible to viral infections, although the occurence is rather rare. The most common is infection with herpes simplex virus (called eczema herpeticum) [188]. Another recently described case is infection with coxsackie virus (called eczema coxsackium) [189]. Atopic dermatitis is usually treated with topical agents, including moisturizers, corticosteroids, calcineurin inhibitors, or antimicrobials [190]. The mode of action is restoring the skin barrier, reducing inflammation, and reducing the bacterial load. Bacteriotherapy has also been tested in the case of atopic dermatitis, through application of coagulase-negative *Staphylococcus* spp. (for instance, *S. epidermidis* and *S. hominis*). In a mouse model, *S. hominis* provided selected protection against *S. aureus* by secreting lantibiotics and showed potential in producing AMPs [191].

### 5.3. Gut Microbiome and Diet Implications in AD

Studies have shown a gut dysbiosis association in patients with atopic dermatitis. The gut microbiome of AD patients was enriched in *Faecalibacterium prausnitzii*, had more genes encoding for release of molecules that can damage the gut epithelium, and had lower levels of butyrate and propionate, which possess anti-inflammatory properties [107]. Higher levels of *Clostridium* and *Escherichia* were found in the gut of atopic infants compared to healthy controls [108,109,110,111]. *Clostridium* and *Escherichia coli* in the intestine can contribute to an inflammatory state [110]. On the other hand, lower levels of *Akkermansia*, *Bacteroidetes*, and *Bifidobacterium* were found in AD patients, compared to healthy controls [112,113]. Butyrate-producing bacteria (f.i. *Coprococcus*) were more abundant in healthy infants or infants with mild AD, compared to infants with severe AD [192]. A likely effective therapeutic option for AD involves the consumption of probiotics, for which a considerable number of studies have been published [193]. In most of the studies *Lactobacillus* and *Bifidobacterium* have been tested [194]. Studies have been conducted in children and adults and during pregnancy, for which often contrasting efficacy results have been obtained [195]. Evidence from a meta-analysis supports the use of probiotics for the treatment of AD in infants; however, the benefit likely results from primary prevention of atopic dermatitis, as also concluded by the World Allergy Organization [114,196,197]. The prophylactic effect of probiotics is likely due to its mediating role on the host immune system. Probiotics can interact with dendritic cells, can balance Th1/Th2 immunity, and can enhance Treg activity, as described in in vitro and in animal models [198,199]. These studies show the impact of the gut microbiome (dysbiosis) on Th2-type immune response to allergens in the skin [200]. Diet has been implicated in atopic dermatitis and Th2-driven inflammations. A reduced consumption of fruit, vegetables, and ω-3 fatty acids and increased consumption of ω-6 fatty acids have been linked to atopic dermatitis [201,202]. Epidemiologic studies have demonstrated associations of atopic dermatitis (and asthma) with margarine, fish, ω-6 polyunsaturated fatty acid (PUFA), and ω-3 PUFA [202]. Further research is nonetheless required to prove the conclusive effect of dietary manipulations on the reduction in atopic disease (and asthma), as previous studies have failed to do so [202,203].

## 6. Psoriasis

### 6.1. Psoriasis Pathophysiology

Psoriasis is an immune-mediated inflammatory disease (IMID) and one of the most prevalent chronic skin diseases (0.1–12%) in the world [204]. It is characterized by red, scaly, and thickened skin lesions that can occur at any site of the body [205]. It is a multifactorial disease with an intimate interplay between genetic susceptibility, lifestyle, and environment [205,206,207,208,209]. Numerous comorbidities are reported, suggesting psoriasis to be a systemic disease rather than just a skin disease [210,211]. Similarly, stress has been reported to be an important trigger and crucial exacerbating factor [212,213]. It is primarily considered a Th17 disease with a major role for IL-23/IL-17-mediated inflammation, where tumor necrosis factor (TNF) enhances the inflammatory feedback loop [214]. Consequently, moderate-to-severe psoriasis is treated with therapeutic antibodies, termed biologics, that target these cytokines, including TNF, IL-17, IL-23, and IL-12/23. These drugs have revolutionized the therapeutic landscape in related IMIDs as well, such as hidradenitis suppurativa (HS), rheumatoid arthritis, and IBD (Crohn’s disease (CD) and ulcerative colitis (UC)). Interestingly, psoriasis is also characterized by a type I interferon (IFN) signature in lesional skin, including upregulated expression of IFN-stimulated response element (IFN-ISRE) genes [215,216,217]. Since psoriasis has no clear cause, it is not considered a classic autoimmune disease. Nonetheless, the AMP LL-37 is found complexed with DNA in increased levels in lesional skin and is targeted by autoantibodies in the arthritis subform of psoriasis [218]. However, the presence of specific cytokine profiles in certain diseases associated with specific antimicrobial responses begs for the role of the microbiome in a disease such as psoriasis, which for instance includes an antiviral response (i.e., type I IFN).

### 6.2. Skin Microbiome in Psoriasis

The psoriatic skin microbiome has been described in several studies [84,85,86,87], and is mainly characterized by a relative higher abundance of *Staphylococcus* and *Streptococcus* species. The various studies often show different outcomes: some studies report a decreased microbial diversity, whereas others describe an increase in diversity. Yerushalmi et al. performed a systematic review on microbial studies in psoriatic disease and reported a general decrease in α-diversity: a higher and lower relative abundance of Firmicutes and Actinobacteria, respectively, in comparison to healthy controls [88]. At the genus level, the results are less consistent according to the systematic review: *Corynebacterium*, *Staphylococcus*, and *Streptococcus* are reportedly more present in lesional skin, whereas a decrease in *Cutibacterium* is observed [88]. This discrepancy supposedly stems from the variety in study design (e.g., lesional skin versus nonlesional skin versus healthy controls, body sites, and medication) and analytic methodology [219]. In psoriasis, a lower abundance of *S. epidermidis* and *C. acnes* may enable increased colonization of *S. aureus* [87]. Indeed, *S. aureus* colonization has been found to stimulate Th17 polarization in mice, suggesting that *S. aureus* triggers IL-17-mediated skin inflammation [87].

A clinical subform of psoriasis, called guttate psoriasis, is usually triggered by a streptococcal throat infection and generally evolves into the vulgaris (plaque) form. People with psoriasis vulgaris also report exacerbation of the disease severity following tonsillitis. Psoriasis associated with tonsillitis may be controlled by tonsillectomy [220,221]. HLA-C*06:02, a well-known psoriasis-associated single nucleotide polymorphism (SNP), has been found to be associated with chronic and recurrent streptococcal tonsillitis [222].

The effect of anti-psoriasis treatments on the skin microbiome has been investigated as well. Psoriasis patiens treated with narrowband UVB light therapy displayed reduced presence of Firmicutes, *Staphylococcus*, *Finegoldia*, *Anaerococcus*, *Peptoniphilus*, *Gardnerella*, *Prevotella*, and *Clostridium* spp. in lesions posttreatment [223]. Conventional and biological systemic treatments (e.g., cyclosporin A, retinoic acids, fumarates, methotrexate, adalimumab, and ustekinumab) resulted in a change in the Actinobacteria to Firmicutes ratio, with biologics having the greatest effect [224].

Characterization of the viral microbiome in psoriasis has not been studied as extensively as its bacterial counterpart. Interestingly, the presence of a type I IFN signature in psoriasis suggests an antiviral response. Triggers through viral infections have been described, albeit evidence remains limited [225,226].

### 6.3. Gut Microbiome and Diet Implications in Psoriasis

People with psoriasis have an increased risk to develop intestinal immune disorders, such as IBD, UC, and celiac disease [118,119,227]. The exact mechanism is not entirely understood, and though many pro-inflammatory cytokines play similar roles in IMIDs, the responses to treatments may differ entirely: the IL-17 blockade is beneficial in psoriasis but rather harmful in IBD [228,229]. Integrity issues in psoriasis are found not only in the skin but also at the intestinal level. Structural aberration in the form of decreased surface in the jejunum was reported in psoriasis patients compared to healthy controls [230]. Other aberrations have been reported as well, including intestinal infiltration of lymphocytes. Lactose intolerance is significantly more present in psoriasis and is even associated with psoriatic severity [227]. Loss of intestinal integrity has been reported in psoriasis based on a 51Cr-labeled ethylenediaminetetraacetic acid (EDTA) absorption test [231] and more recently through increased levels of barrier-related proteins such as claudin-3 and intestinal fatty acid binding protein (I-FABP) in serum [232]. Higher levels of fecal calprotectin were found, were correlated to disease severity [233], and were especially increased when joint inflammation was involved [233]. Studies have shown the presence of ribosomal DNA in the peripheral blood of psoriasis patients, including DNA from *Streptococcus* and *Staphylococcus* spp. [234,235]. In mice, intestinal inflammation drove imiquimod-induced psoriasis-like skin inflammation [116].

A number of studies investigated the gut microbiome of psoriasis patients, which showed differences in β-diversity (Table 2). Two studies reported lower relative abundance of Bacteroidetes and higher Firmicutes in psoriasis patients compared to healthy controls [115]. The gut microbiome has also been investigated in response to anti-psoriasis treatment: secukinumab, an IL-17 inhibitor, had a greater impact on the gut microbiome in comparison to ustekinumab, an IL-12/23-p40 inhibitor. In detail, the relative abundance of Proteobacteria, Pseudomonadaceae, Enterobacteriaceae, and Pseudomonadales increased in response to secukinumab, whereas Bacteroidetes and Firmicutes declined [120]. These findings suggest a link between psoriasis and the intestinal health.

Lifestyle has major implications in psoriasis: smoking and alcohol have been associated with the exacerbation of skin lesions and even suboptimal responses to treatments, whereas obesity is an independent risk factor for the development of psoriasis [236]. A healthy weight is associated with beneficial effects: HFDs are associated with the exacerbation of psoriasis, whereas weight reduction had a positive outcome on psoriasis severity [237,238,239]. A response to treatment may also be susceptible to dietary intake, as a very low-calorie ketogenic diet has been shown to improve response in patients with a psoriasis relapse [237,240]. Intermittent fasting according to the Ramadan regimen has also been found to positively impact moderate-to-severe psoriasis [241]. Indeed, the intestinal dysbiosis in patients with psoriasis has been the target for probiotics [242]. A mixture of strains was tested in psoriasis and found to be beneficial, up to 6 months after intervention, with fewer relapses in the group treated with the probiotic mixture [243]. Recently, an oral derivative of a single strain of *Prevotella histicola* was tested for psoriasis. In the murine imiquimod model, it was found to be effective, which was confirmed in a phase 1b trial in humans, yet the results remain to be published [122,244].

## 7. Hidradenitis Suppurativa (HS)

### 7.1. HS Pathophysiology

Another chronic cutaneous IMID is hidradenitis suppurativa, with a global prevalence of 0.3% [245]. HS is characterized by occlusion of the apocrine glands, though it is also commonly known as acne inversa, as it occurs in the inverse areas such as the axillae, inframammary regions, groin, and genital and perianal regions. It typically involves recurring, draining, and inflamed lesions that are painful and disfiguring. The lesions consist of chronic subcutaneous sinus tracts, cutaneous fistulae, or dermal-cutaneous scars. Though its etiology remains incompletely understood, research reports a dysregulation of inflammatory cytokines and occlusion of the follicles. TNF is considered a key cytokine, orchestrating the inflammatory loop with VEGF, IL-8, and IL-1β, whilst skin biopsies show an increased ratio of Th17 cells compared to Tregs [246,247]. The use of adalimumab, an anti-TNF antagonist, was found to be effective in the treatment of HS and to normalize the Th17/Treg population. It is a multifactorial disease, where lifestyle plays a significant role, including exacerbating effects of smoking and obesity. It remains incompletely understood what the exact underlying mechanism is, and therapeutic interventions, additionally to anti-TNF, include laser-assisted hair removal and antibiotics, yet no cure exists.

### 7.2. Skin Microbiome in HS

HS lesions present with bacterial infections, which are clinically considered secondary. However, the lesions exhibit a distinct cutaneous microbiome in lesional and nonlesional HS skin in comparison to healthy controls. More specifically, anaerobic species were found in lesions, e.g., *Prevotella* and *Porphyromonas*, whereas aerobic commensals were reduced. Interestingly, the HS-microbiome may have clinical relevance as *Fusobacterium* and *Parvimonas* spp. were found to correlate to disease severity [89]. In addition, *Saccharomyces cerevisiae* yeast and one of its wall components, mannans, may also play a role in HS: anti-*Saccharomyces cerevisiae* antibodies (ASCAs) have been found in HS-serum and to be specifically present in comparison to psoriasis vulgaris and healthy controls, underlining its importance as a biomarker [90]. Assan et al. even reported a significant elevation in the severe cases (Hurley III stage), suggestive for a prognostic marker for disease severity [90].

### 7.3. Gut Microbiome and Diet Implications in HS

The main link with the gut is based on the increased risk in developing CD and UC: based on a systematic review, usually a two-fold odds ratio was found [248]. Moreover, both diseases respond to anti-TNF treatment, suggesting similar inflammatory pathomechanisms. Especially the presence of perianal fistulae in CD is interesting, and molecular research of HS and CD fistulae has found that CD161+ T lymphocytes are enriched in these lesions, which can differentiate into pathogenic Th17 cells [249]. Interestingly, the presence of ASCAs in HS can be linked to several intestinal disorders such as Crohn’s and celiac disease, where ASCAs are prevalent as well. Positivity for ASCA suggests a systemic response to the oligomannosidic epitopes of yeast. Such observations imply that systemic tolerance to microbial antigens may be exhibited in tissue-specific manifestations, including cutaneous (HS) and intestinal (Crohn’s and celiac disease) [250]. In HS, several lifestyle factors such as smoking, alcohol, and obesity are considered exacerbating factors. Especially cessation of smoking has been associated with significant improvement in HS lesions [251,252].

Based on the intimate link with Crohn’s disease, dietary interventions have been suggested as clinical interventions for HS. However more importantly, obesity is also a known independent risk factor for HS, and a weight loss of at least 15% has been associated with diminished disease severity [253]. Imbalance in the gut microbiome has been associated with diets high in fat, including an increment in Firmicutes and a reduction in Bacteroidetes. How the bacterial HS-associated gut microbiome responds to low fat diets remains to be elucidated. Avoidance of food containing or made with the yeast *S. cerevisiae* has resulted in promising long-term results in HS, including a reduction in inflammation and amelioration of clinical response to (surgical) treatments [254]. Though other “avoidance” diets are suggested for the treatment of HS, randomized controlled trials are lacking to provide solid evidence for HS [255].

## 8. Rosacea

### 8.1. Rosacea Pathophysiology

Rosacea is a chronic inflammatory dermatosis characterized by various skin lesions predominantly on the face including erythema, papulopustules, telangiectasia, and/or ophthalmic involvement that affects up to 15% of the Caucasion population with fair sun-sensitive skin (skin phototypes I and II) [256,257,258]. Although the pathophysiology of rosacea remains unclear, neurovascular dysregulation, impaired immunity, external factors, and genetic inheritance are suggested to play important roles in the disease progression [92]. Rosacea patients retain a dysregulated innate immune system that causes an abnormal inflammatory cytokine release and an AMP response. Cathelicidin expression is significantly increased in the epidermis of rosacea affected skin compared to normal skin [259,260]. In granular or cornified layers of normal skin, cathelicidin nis early absent whereas its expression is greatly induced by wounding or infection [261]. LL-37 is the most common ly found cathelicidin peptide in rosacea patients [260]. Toll-like receptor 2 (TLR2) levels are increased in rosacea patients and stimulate KLK5 [262]. In addition, particular cathelicidin types stimulate and control leukocyte chemotaxis, vasodilation, angiogenesis, and the expression of extracellular matrix proteins [263,264,265]. Neurogenic inflammation might also play an important role in the pathogenesis of rosacea. Various rosacea triggers, including heat and dietary factors, might activate and upregulate the transient receptor potential (TRP) ion channels of vanilloid type (TRPV), which are expressed by sensory nerves as well as by keratinocytes [266]. The TRP channels might be targets for rosacea patients, as they play a role in inflammation, pain perception, and vasoregulation [262,266].

### 8.2. Skin Microbiome in Rosacea

The skin of rosacea patients regularly contains an overgrowth of commensal skin microorganisms. Higher concentrations of *Demodex folliculorum* were detected, which usually inhabit the sebaceous glands. The *D. folliculorum* mite density has been reported to attain up to 10.8/cm${}^{2}$ in rosacea patients in comparison to 0.7/cm${}^{2}$ in controls [91]. TLR2 is activated by cell-membrane components of the *Demodex* mite, which triggers KLK5 activity [267]. The use of permethrin against *D. folliculorum* reduced the mite abundance; however, the skin lesions did not recover [268]. For this reason, researchers proposed bacteria as a causative agent for the inflammatory responses in rosacea, and the role of *Bacillus oleronius* and *Staphylococcus epidermidis* have been investigated [262,267,269,270]. The study of Woo et al. analyzed the influence of oral antibiotics on the composition and diversity of the skin microbiome in rosacea patients. *Staphylococcus epidermidis*, a skin commensal, is the predominant species, followed by *C. acnes*. Rosacea severity increased with age and the relative abundance of *C. acnes* decreased, whereas the relative abundance of *Snodgrassella alvi* increased. *Geobacillus* and *Gordonia* were significantly associated with rosacea severity [271]. The use of topical metronidazole (1% cream) did not alter the skin microbiota composition [272]. Zaidi et al. described that oral doxycycline (100 mg for 6 weeks) did not affect the α-diversity but demonstrated an increase in the relative abundance of *Weissella confusa* [273]. In contrast, Woo et al. reported a decrease in *W. confusa* [271]. Further studies are thus needed to assess the effect of oral antibiotics on the skin microbiome composition.

### 8.3. Gut Microbiome and Diet Implications in Rosacea

A link between gut microbial dysbiosis and rosacea has been hypothesised, as there is an increased risk of gastrointestinal disorders in rosacea patients [274]. Especially *Helicobacter pylori* infection (HPI) has been associated with the disease [123]. The prevalence of small intestinal bacterial overgrowth (SIBO) is increased in rosacea patients. The elimination of SIBO resulted in a significant reduction in cutaneous lesions [124]. A population-based cohort study with 50,000 Danish rosacea patients could identify a higher prevalence of celiac disease, CD, UC, HPI, SIBO, and irritable bowel syndrome (IBS) among the rosacea subjects compared to the control subjects [274,275]. However, comprehensive studies are missing that describe the role of gut dysbiosis in rosacea. A recent Korean study found a link between several enteral microbiota and rosacea in a group of 12 female subjects with rosacea [125]. The abundance of enteral microbiota was similar between patients with rosacea and rosacea-free controls yet differed in composition. A higher abundance of *Acidaminococcus* and *Megasphaera* and a lower abundance of Peptococcaceae and *Methanobrevibacter* were reported [125]. The study of Chen et al. demonstrated a reduction in the fecal microbial richness in rosacea patients as well as a distinct fecal microbial community. The altered microbial composition might be due to sulfur metabolism, cobalamin, and carbohydrate transport [276]. The microbiome might be a critical therapeutic target. An important note is that there is a lot of interindividual variability in the human intestinal microbiome composition. Several reasons may account for this variability, including genetics, environmental exposure, hygiene, geography, ethnicity, etc. [277].

Rosacea exacerbations are frequently linked to dietary factors that can mainly be categorised into heat-related, alcohol-related, capsaicin-related, and cinnamaldehyde-related [278]. There are multiple proposed mechanisms of action (MOA); one of them is through the activation of TRP channels [279]. A second MOAPlease define if appropriate. is through the gut–skin connection [274].

## 9. Dandruff and Seborrheic Dermatitis

### 9.1. Dandruff and Seborrheic Dermatitis Pathophysiology

Dandruff is a skin condition that mainly affects the scalp, resulting in skin flaking and pruritus. It occurs in 30–50% of the world’s population, with males generally more affected than females [280,281]. Severe forms of dandruff include inflammation of the skin and are known as seborrheic dermatitis. Seborrheic dermatitis is a chronic and inflammatory dermatosis with recurrent character, and its pathophysiology is very similar to that of dandruff [282]. The exact causes of these conditions remain unknown. However, several factors have been implicated in the progression of the skin conditions, including sebum levels, immune response, stress, environmental and hormonal changes, and individual sensitivity [283]. Moreover, seborrheic dermatitis has been linked and can be caused by an inflammatory immune response to *Malassezia* spp. [284]. It is usually treated with anti-dandruff shampoos, containing antibacterial and antifungal agents.

### 9.2. Skin Microbiome in Dandruff and Seborrheic Dermatitis

Dandruff and seborrheic dermatitis are generally associated with a fungal component. *Malassezia* spp. are lipophilic and dominant fungi colonizing the human scalp and are the most abundant yeast species of the skin mycobiome [280]. *Malassezia restricta*, *Malassezia furfur*, and *Malassezia globosa* are the most abundant species of the *Malassezia* genus. An inflammatory reaction to excess *Malassezia* spp. growth on skin has been associated with seborrheic dermatitis [93]. *Malassezia* spp. are thought to cause an overproduction of oleic acid, which disturbs the stratum corneum cells and evokes an inflammatory response on the scalp [93]. This results in irritating free fatty acids and other metabolites, which can lead to more sebaceous secretions on the scalp, which in turn leads to an inflammatory response that results in skin changes [285]. Dandruff and seborrheic dermatitis occur solely on skin areas with high levels of sebum [93]. Preferencial sites are sebaceous gland-rich areas such as the face, ears, scalp, and upper trunk. Patients with oily skin are prone to developing seborrheic dermatitis [94]. Analysis of the *M. globosa* genome showed an absence of fatty acid synthase, while many lipase and phospholipase genes/enzymes were present and active on human scalp [93]. This explains the nature of this yeast to heavily rely on external (sebaceous) fatty acids to survive. A bacterial impact was also suggested, with an imbalance in *Cutibacterium* and *Staphylococcus* species [95,96].

### 9.3. Gut Microbiome and Diet Implications in Dandruff and Seborrheic Dermatitis

The link between gut dysbiosis and dandruff/seborrheic dermatitis has been controversial. Some deviations have been detected in the intestinal mucosa of patients with seborrheic dermatitis [286]. A clinical study on probiotics consumption (*Lactobacillus paracasei* strain) found significant improvements in severity and symptoms of moderate to severe dandruff compared to a placebo treatment [126]. However, the influence of the gut microbiome composition on seborrheic dermatitis and dandruff remains to be elucidated.

Diet has been reported as having an important contribution in the production of sebum. Dietary lipids, glucose intake, and acetate have been indicated as influencing for sebaceous gland activity [287]. Sugar consumption is often higher in patients with seborrheic dermatitis, compared to healthy control groups [288]. Caloric restrictions have been linked to reduced sebum production [289]. Increased levels of vitamin A in the blood has also been linked to a decreased sebum production [290]. Dandruff patients are often advised to avoid sugar, animal fats, and greasy food products and instead consume more vegetables, water-based fruits, seeds, fish, biotin, and vitamin B, although no conclusive evidence has been found for such recommendations [291].

## 10. Alopecia

### 10.1. Alopecia Pathophysiology

Alopecia areata is a skin condition with a prevalence of 2% and is clinically characterized by small areas of hair loss on the scalp and/or all over the body [292]. The pathophysiology is still unclear but there is some strong evidence that autoimmune reactions cause inflammations at the site of the hair follicle. Research indicates that different cells of the innate and adaptive immune system are correlated to alopecia areata. Th cells, cytotoxic T cells, natural killer cells, and DCs are present at the hair follicle during the anagen (growth) phase of the hair. The autoimmune responses of these cells cause the production of cytokines such as IFN-γ and TNF-α, which leads to collapse of the hair follicle [293]. The factors that cause this immune response remain unknown. However, there is some evidence that genetic disposition, several environmental factors, and even maybe the skin microbiome can have some influences on the disease as well [294].

### 10.2. Skin Microbiome in Alopecia

The scalp microbiome mainly consists of Corynebacteriaceae, Propionibacteriaceae, and Staphylococcaceae [99]. A small fraction of the scalp microbiome also consists of fungi, with *Malassezia restricta* being the most important one. These microorganisms have a symbiotic relationship on healthy scalp, while dysbiosis can cause pathological conditions. A higher abundance of pathogenic taxa in the hair follicle can lead to infections and can contribute to a pro-inflammatory state on the scalp [295]. Analysis of the scalp microbiome of patients with alopecia areata demonstrated an increase in *C. acnes* in combination with a decrease in *S. epidermidis* [99]. A disbalance in *Cutibacterium*/*Staphylococcus* spp. can potentially play a role in alopecia areata [99]. An increase in cytomegalovirus and *Alternaria* fungi in alopecia areata has also been postulated [97,98]. Skin microbiome data on this scalp condition remains nonetheless scarce.

### 10.3. Gut Microbiome and Diet Implications in Alopecia

An association between gut dysbiosis and alopecia areata has been considered. Genes that are related to alopecia areata may also affect gut colonization with microorganisms that induce a Th1 response, which leads to the production of IFN-γ, as IFN-γ signals through a JAK/signal transducer and activator of transcription (STAT) signal pathway [293]. Induction of this pathway can cause abnormal growth of hair follicle cells and can even progress into hair loss. Furthermore, dysbiosis of the gut microbiome provokes other diseases through manipulation of the T cell activity near and distant to the site of induction [296]. A case report revealed hair growth in two patients with alopecia areata who were treated with a fecal microbiota transplant (FMT) [128]. This also supports the hypothesis of the potential role of the gut microbiome in the pathophysiology of alopecia areata. Some gut bacterial differences were identified in alopecia areata patients, without major differences [127]. Based on the limited studies found in literature, a clear association between gut dysbiosis and alopecia areata has not yet been determined.

A nutrient deficiency can impact hair growth and structure. Metal deficiencies, such as iron and zinc, can cause hair loss. Low serum ferritin and zinc are more prevalent in patients with alopecia areata. Vitamin deficiencies can also result in hair loss. Niacin and biotin deficiency are proven to cause alopecia areata. Vitamin D takes part in hair follicle cycling and vitamin A activates hair follicle stem cells. [297]. These deficiencies are linked to hair loss and/or alopecia areata; however, limited information is available on the effect of supplement intake and its association with hair loss and alopecia areata [297]. Furthermore, some diet alterations might also benefit the hair growth of alopecia areata patients [298]. A gluten-free diet stimulated the hair growth of patients suffering from celiac disease [298]. People who followed a soya-based eastern diet have a decreased risk for alopecia areata, less than 1% instead of a global +/−2% risk [293]. The Mediterranean diet, rich in raw vegetables and fresh herbs, or a high protein diet is a potential treatment for alopecia [298]. However, the effect of a food limitation as a treatment for alopecia areata needs to be further explored.

## 11. Skin Cancer

### 11.1. Skin Cancer Pathophysiology

Skin cancer is a common malignancy and can be divided into two categories: invasive melanoma, in which melanocytes divide uncontrollably, and non-melanoma skin cancers (NMSCs). The latter covers tumors with keratinocytic origin such as basal cell carcinoma (BCC) and squamous cell carcinoma (SCC) [299]. There is a wide variety of risk factors that may lead to melanoma and NMSCs, which includes constitutional predisposition, immunosuppressive status, and exposure to environmental risk factors such as ultraviolet radiation [300]. In addition, actinic keratosis and Bowen’s disease may also result in SCC [301]. During the last decennia, immunology involving cutaneous components was better understood by the immunosurveillance mechanisms and the immunoediting framework. The immunogenicity of tumor cells changes by an altered expression of (tumor-associated) antigens, such as reduced MHC-1 expression, resulting in the development of malignancy [302].

### 11.2. Skin Microbiome in Skin Cancer

The impact of viruses and UV radiation on skin cancer has already been extensively examined. Recently, a lower incidence of skin cancer was discovered in germ-free rats. As a result, it is hypothesized that dysbiotic skin microbiota can result in the development of several skin cancers. However, it remains unclear whether tumor cells or microbial dysbiosis trigger progression [303]. A number of studies have explored the link between several skin cancers and dysbiosis of the bacterial skin microbiome in inflammatory diseases involving Th17, such as psoriasis and acne [103]. Moreover, SCC and actinic keratosis have recently also been associated with an increase in certain strains of *S. aureus* in combination with a decrease in skin commensals [100]. Cheng et al. associates the latter with the development of BCC [101]. Additionally, melanoma samples showed increased levels of *Fusobacterium* and *Trueperella* genera according to a recent study by Mrázek et al. [102]. Furthermore, an increase in Merkel cell polyomavirus (MCPyV), a virus thought to be a persistent resident of the skin, can lead to Merkel cell carcinoma (MCC) [103]. On the other hand, specific *S. epidermidis* strains were shown to selectively inhibit proliferation of tumor cell lines as it protects the progression of UVB-induced skin papillomas in preclinical models [304]. *S. epidermidis* produces 6-N-hydroxyaminopurin, which interferes in *Streptococcus* synthesizing DNA polymerase without interfering primary keratinocyte growth [303]. Protective reactive oxygen species (ROS) are reduced in actinic keratosis and BCCs [165,305]. This shows that skin commensals, such as *C. acnes* and *S. epidermidis*, can protect the host from UV-induced DNA damage. Furthermore, treatments with topical probiotics are suggested to reduce the risk of skin cancer due to increased immune surveillance and reduced chronic inflammation. In fact, topical probiotics may alter the tumor microenvironment by changing the immune responses, which may lead to therapeutic effects [103]. However, more research is still needed to fully understand the role of skin microbiota in skin cancer.

### 11.3. Gut Microbiome and Diet Implications in Skin Cancer

Cancer patients are also frequently subjected to dysbiotic gut microbiota because of therapies affecting the composition and immunity of these microbiota [306]. Although the link between this dysbiosis and skin cancer in particular remains unclear, the link with cancer in general has already been investigated to a limited extent. For example, colorectal cancer (CRC) is associated with an increase in *Bacteroides fragilis* in murine models. Additionally, an altered gut microbiome leads to an increased risk to develop CRC [129]. Moreover, Guo et al. discovered the link between *Helicobacter pylori* and an increased risk of pancreatic cancer [130]. This may be a consequence of a disturbed gut microbiome, which has been described in *Helicobacter*-associated diseases [131]. However, more research is needed to investigate the correlation between gut dysbiosis and skin cancer. Finally, it is uncertain whether tumor development is secondary to bacterial dysbiosis.

## 12. Wound Healing

### 12.1. Wound Pathophysiology

Cutaneous wound healing is a very complex and organized process and consists of overlapping phases of acute healing [307]. Multiple cell types, primarily epidermal keratinocytes, neutrophils, and macrophages, are involved and interact with residing commensal microbiota. The latter one may colonize wounds and may stimulate wound healing by promoting the innate immune system. Subsequently, keratinocytes expand and migrate, fibroblasts migrate and amass the extracellular matrix (ECM), and angiogenesis ensues during the proliferation phase. In the remodelling phase, the ECM restores, scar formation appears, and the epidermal skin barrier recovers [307]. When one of these phases is hampered, the epithelial barrier will not heal properly and a wound becomes chronic. Impaired wound healing is a major challenge for the health care system, as it affects roughly 1% to 2 % of the population in developed countries [308,309]. The prevalence of chronic wounds is higher in older people with underlying pathologies including diabetes mellitus, vascular disease, and obesity [310]. The cellular programs restoring the skin barrier do not function properly in chronic wounds [307,311,312]. Impaired wound healing is characterized by accelerated keratinocyte proliferation, impaired migration, and fibrosis. In addition, several processes such as angiogenesis, ECM remodelling, and induction of stem cells are hindered. Chronic wounds were also continuously inflamed, as was demonstrated by several studies [307,313,314,315,316,317]. However, the underlying cellular and molecular mechanisms of impaired wound healing are not yet fully understood. Especially the role of skin microbiome in impaired wound healing and the application of antimicrobial products are still questioned [312].

### 12.2. Wound Skin Microbiome

Wounds provide an ideal opportunity for microbiota to obtain access to underlying tissues and to meet the ideal conditions to colonize and grow [318,319]. Commensal microbiota are thought to be beneficial for the wound healing process. They are essential for regulating the skin innate immune system, as they stimulate the production of antimicrobial molecules that provide protection against intracellular pathogens [318,320,321]. Keratinocytes are effective killers of intracellular bacteria in a perforin-2 (P-2)-dependent manner [322]. Skin commensal bacteria, such as *S. epidermidis*, are able to regulate the gamma delta (γδ) T cells and to induce the P-2 expression. Intracellular *S. aureus* were destroyed by skin cells as a result of the increased P-2 expression, which was induced by *S. epidermidis* [323]. In addition, certain *S. epidermidis* strains produce trace amines that accelerate wound healing in mice [324]. Disruption of the normal skin microbiota may contribute to impaired wound closure and the chronic wound pathology. Wound microbiome studies reveal that 21 bacterial families account for the majority of microbiota that colonize chronic wounds [325,326]. Methicillin-resistant *S. aureus* (MRSA) is one of the most common pathogens to colonize wounds [104]. Similarly, biofilm forming bacteria have been associated with delayed wound healing [326]. Probiotics, like *Lactobacillus* as well as fermented products have been tested to counteract the detrimental effects of wound colonizing microbes [104,105].

### 12.3. Gut Microbiome and Diet Implications in Wound Healing

Alterations in the commensal skin microbiome may contribute to the formation of chronic wounds. Recent research in animal models suggests that probiotics may hinder and cure non-healing wounds. Kefir extracts in topical gels have improved the epithelialization and the collagen generation in burn injuries in rats compared to controls that were treated with silver sulfadiazine [327]. The administration of oral probiotics to ultraviolet injured mice modulated the quantity of immune cells in the skin as well as the IL-10 levels, illustrating the immunomodulating potential of probiotics in skin tissues [46]. Supplementation of lactic acid bacteria in drinking water stimulated the healing process in mice compared to controls. In addition, the probiotic strain *Lactobacillus reuteri* improved wound healing by stimulating oxytocin, which induced the CD4 + Foxp3 + CD25 + Treg lymphocytes that convey the wound healing capacity [328]. These data support the notion that Tregs have the potential to modulate the immune system beyond the gut.

## 13. Conclusions

The skin diseases as discussed in this manuscript result from a complex interaction between genetic susceptibility, lifestyle, and the immune system. More specifically, the latter is in constant orchestration with the nervous and endocrine systems. These interactions allow microbiota to play a key role, especially in organs such as the skin and gut that are richly perfused with immunoregulators and microbiota. Additionally, observations such as the prevention of AD through probiotics and the increased prevalence of intestinal comorbidities in chronic skin diseases suggest that skin diseases can be linked to the gastrointestinal system. The main hypothesis relies on the gut health, which is directed by dietary factors, mediated through the intestinal microbiome and the immune system, leading to systemic effects, including skin health. The integrity of the intestinal barrier plays a key role, which if compromised, leads to a “leaky gut”—an impaired intestinal barrier. However, its existence remains heavily debated.

This intestinal microbial dysbiosis poses an interesting field of investigation and applications. Pre- and probiotics aimed at the intestinal microbiome may be used for targeting skin health [45]. Probiotic-fed mice with *Lactobacillus reuteri* demonstrated a shinier and thicker fur mediated through IL-10 and, upon the addition of purified Foxp3+ T cells, also improved the integumentary system [329]. In a placebo controlled human study, healthy volunteers obtained a lower transepidermal water loss and lower skin sensitivity when consuming probiotics compared to the placebo group [330]. Interestingly, consumption of probiotics or live bacteria that are beneficial for the gastrointestinal system has potential to prevent and manage various skin diseases, such as acne vulgaris, atopic dermatitis, and psoriasis [331,332,333,334,335,336]. Similar beneficial health effects have been determined by consuming prebiotics and synbiotics [337,338]. However, specific diets such as caloric restriction and low fat diets have also been associated with improved intestinal epithelial barrier or with cutaneous improvements, including acne vulgaris, AD, psoriasis, wound healing, skin cancer, and even skin aging [339,340].

The attractiveness of targeting the gut microbiome through oral delivery seems inversely correlated with the complexity of targeting the gut–skin axis: modulation of the intestinal microbiome may lead to systemic effects, including the skin and other organs. Properly designed clinical trials are needed to assess the effectiveness of microbiome-targeted treatments. Recent advances in short-read and long-read sequencing technologies permit detailed understanding in gut and skin microbial mediators. These technologies should be accompanied by biomarker analyses (f.i. IgA, calprotectin, and immune measurements) to identify the interplay between the microbiome and the gut barrier integrity.

Though the microbiome also includes viral microbiota, little evidence is available on how viruses impact skin diseases and the intestinal health. The upcoming whole genome sequencing should facilitate our knowledge on the role of viruses in the gut–skin axis.

Skin health outcomes need to be defined in order to determine the impact. Skin tape stripping, which has recently been introduced for protein and mRNA quantification, will enable noninvasive sampling, which is less burdensome for the study subjects. However, one should take into account that skin tape stripping only reveals limited information in comparison to skin biopsies.

Lastly, the study protocols need to take this complexity into account: the quality of recording dietary habits is crucial and relies on the chosen method, such as patient-reported outcome measures (e.g., a food frequency questionnaire versus digital apps for calorie counting and food databases). Moreover, the moment of sampling should be carefully considered: study subjects may have fasted at the moment of sampling by skipping breakfasts versus those who did not. Moreover, the impact of the circadian rhythm needs to be incorporated into future clinical trials. The current literature lacks studies reporting on the impact of the circadian rhythm of nutrition uptake. The study of Parkar et al. reviewed current evidence that demonstrated the effect of altered sleep and eating patterns that may disrupt the host circadian system and that affect the gut microbiome. They concluded that a distortion of microbiome rhythms might at least partly be responsible for an increased risk of obesity and metabolic syndrome linked with insufficient sleep and circadian misalignment [341]. In addition to our own circadian rhythm, the microbiota has also been described to exhibit an internal clock regulated through microbial metabolites [342,343,344]. Presumably, certain nutritional components are better metabolized at certain time points during the day. Indeed, high caloric intake during evening hours has been associated with weight gain, whereas the same caloric intake during morning hours results in weight maintenance [345].

In conclusion, the gut–skin axis, with a central role for our microbiota, poses an exciting field of research, with promising therapeutic and cosmetic applications. Dissecting the interactions between the microbiome and the hosting tissues will lead to a better understanding of health and disease and will create novel opportunities. The need for well-designed trials is primordial and will require multidisciplinary teams to work together, reflecting the cooperation between our own bodies and microbiota.

## Figures and Tables

**Table 1:** Skin microbiota associated with nine common skin disorders.

Disease	Associated Skin Microbiota	Additional Remarks	Reference
1. Acne vulgaris	Particular *C. acnes* strains	Administered probiotic bacteria could play a protective role.	[71,72,73,74,75,76,77,78,79]
2. Atopic Dermatitis	Decreased bacterial diversity. Increased abundance of *S. aureus*.	Herpes simplex virus and coxsackie virus can infect AD * skin.	[55,80,81,82,83]
3. Psoriasis	Higher abundance of *Staphylococcus* and *Streptococcus*.	Anti-psoriasis treatments lead to skin microbial changes.	[84,85,86,87,88]
4. Hidradenitis suppurativa	*Saccharomyces cerevisiae*(yeast), *Prevotella*, and *Porphyromonas* (bacteria)	Anaerobic species in lesions.	[89,90]
5. Rosacea	*Demodex folliculorum*(mites)	*C. acnes* decreased and *Snodgrassella alvi* increased. *Geobacillus* and *Gordonia*.	[91,92]
6. Dandruff and Seborrheic dermatitis	*Malassezia* spp. (yeast)	Potential bacterial imbalance.	[93,94,95,96]
7. Alopecia areata	Limited data. Possible imbalance *C. acnes*/ *S. epidermidis*.	Potential role of cytomegalovirus and/or *Alternaria* fungi.	[97,98,99]
8. Skin cancer	Merkel bell Polyomavirus, *Fusobacterium*, and *Trueperella*, *S. aureus*.	Increase in certain strains of *S. aureus* in combination with a decrease in skin commensals can be associated to SCC * or BCC *, and that in MCPyV * can be associated to MCC *.	[100,101,102,103]
9. Wound healing	*S. aureus* and biofilm-forming bacteria.	Lactobacilli and fermented products can be beneficial.	[104,105]

* Abbreviations: Atopic dermatitis (AD), basal cell carcinoma (BCC), hidradenitis suppurativa (HS), Merkel cell carcinoma (MCC), Merkel cell Polyomavirus (MCPyV), and squamous cell carcinoma (SCC).

**Table 2:** Gut microbiota associated with nine common skin disorders.

Disease	Associated Gut Microbiota	Additional Remarks	Reference
1. Acne vulgaris	Decrease in Firmicutes and increase in Bacteroides.	Distinct gut microbiome composition and decreased diversity.	[106]
2. Atopic Dermatitis	Higher levels of *Faecalibacterium prausnitzii*, *Clostridium*, and *Escherichia* (in infants). Lower levels of *Akkermansia*, *Bacteroidetes*, and *Bifidobacterium*.	Probiotics consumption can prevent AD *.	[107,108,109,110,111,112,113,114]
3. Psoriasis	Changes in β-diversity. Gut microbiome changes in reaction to biologicals.	Increased risk of intestinal immune disorders. Diet and gut microbiome can have an impact on inflammation.	[115,116,117,118,119,120]
4. Hidradenitis suppurativa	Unknown	Increased risk in developing CD * and UC *.	[121,122]
5. Rosacea	Can be associated with SIBO *. *Acidaminococcus* and *Megasphaera* increase and *Peptococcaceae* and *Methanobrevibacter* decrease.	Can be associated with *H. pylori* infection.	[123,124,125]
6. Dandruff and Seborrheic dermatitis	Unclear	Probiotic consumption can alleviate moderate to severe dandruff	[126]
7. Alopecia areata	No major differences	FMT * in 2 patients showed restoration of hair growth	[127,128]
8. Skin cancer	Not reported	Other cancers are associated with microbial dysbiosis	[129,130,131]
9. Wound healing	Not reported	Not reported	

* Abbreviations: atopic dermatitis (AD), Crohn’s disease (CD), fecal microbiota transplant (FMT), hidradenitis suppurativa (HS), small intestinal bacterial overgrowth (SIBO), and ulcerative colitis (UC).

**Table 3:** Molecules with potential a modulatory effect on skin and gut either directly or indirectly.

Molecule	Documented/Possible effect in gut	Documented/Possible effect on skin	Reference
**Bacterial metabolites**			
SCFAs *	Anti-inflammatory effects	Anti-inflammatory effects	[132]
Vitamin D	Suppress inflammation in IBD*	Not reported	[133]
Urocanic Acid	Suppress inflammation in IBD*	Not reported	[134]
GABA *	Neurotransmitter modulation	Itch restriction	[135,136]
Dopamine	Neurotransmitter modulation	Inhibition of hair growth	[135,137]
Serotonin	Neurotransmitter modulation	Melatonin modulation	[135,138]
Acetylcholine	Neurotransmitter modulation	Barrier function	[135,139]
Phenol and **p-cresol**	Biomarker of gut dysbiosis	Impaired epidermal barrier function	[140]
**Dietary components**			
Catechins	Anti-inflammatory effects	Anti-inflammatory effects	[141]
Polyphenols	Anti-inflammatory effects	Anti-inflammatory effects	[142]
Lycopene	Selectively utilized by host microbiota	Protection against photodamage	[143,144]
Prolamin	Not reported	Protection against AD *	[145]
Phytomolecules	Not reported	Anti-ageing	[146]
Gluten	Coeliac disease	Skin Rashes	[58,59]

* Abbreviations: Atopic dermatitis (AD), gamma-aminobutyric acid (GABA), inflammatory bowel disease (IBD), and short chain fatty acids (SCFAs).

## Abbreviations

The following abbreviations are used in this manuscript:

AD	Atopic Dermatitis
AMP	Antimicrobial Peptide
ASCA	Anti-*Saccharomyces cerevisiae* Antibody
BCC	Basal Cell Carcinoma
CD	Crohn’s Disease
CRC	Colorectal Cancer
DC	Dendritic Cell
EC	Epithelial Cell
EDTA	Ethylenediaminetetraacetic Acid
FMT	Fecal Microbiota Transplant
GABA	Gamma-Aminobutyric Acid
γδ	Gamma Delta
GI	Gastrointestinal
HFD	High-Fat Diet
HPI	*Helicobacter pylori* Infection
HS	Hidradenitis Suppurativa
IBD	Inflammatory Bowel Disease
IBS	Irritable Bowel Syndrome
IEC	Intestinal Epithelial Cell
I-FABP	Intestinal Fatty Acid Binding Protein
IFN	Interferon
IFN-ISRE	IFN-Stimulated Response Element
IgE	Immunoglobulin E
IGF	Insulin-like Growth Factor
IL	Interleukin
ILC	Innate Lymphoid Cell
IMID	Immune-Mediated Inflammatory Disease
JAK	Janus Kinase
KLK5	Kallikrein 5
LC	Langerhans Cell
LL-37	Cathelicidin AMP
M	Membranous
MAMP	Microbial-Associated Molecular Pattern
MCPyV	Merkel Cell Polyomavirus
MCC	Merkel Cell Carcinoma
MOA	Mechanism of Action
MRSA	Methicillin-resistant *S. aureus*
NMSC	Non-Melanoma Skin Cancer
P-2	Perforin-2
PUFA	Polyunsaturated Fatty Acid
ROS	Reactive Oxygen Species
SCC	Squamous Cell Carcinoma
SCFA	Short Chain Fatty Acid
SIBO	Small Intestinal Bacterial Overgrowth
SREBP-1	Sterol Regulatory Element-Binding Protein 1
STAT	Signal Transducer and Activator of Transcription protein
TLR2	Toll-like Receptor 2
Th	Helper T cell
Treg	Regulatory T cell
TRP	Transient Receptor Potential
TRPV	Transient Receptor Potential Ion Channels of Vanilloid Type
UC	Ulcerative Colitis
UVB	Ultraviolet B
VEGF	Vascularendothelial Growth Factor
